# Signal Differentiation of Moving Magnetic Nanoparticles for Enhanced Biodetection and Diagnostics

**DOI:** 10.3390/bios15020116

**Published:** 2025-02-17

**Authors:** Kee Young Hwang, Dakota Brown, Supun B. Attanayake, Dan Luu, Minh Dang Nguyen, T. Randall Lee, Manh-Huong Phan

**Affiliations:** 1 Laboratory for Advanced Materials and Sensors, Department of Physics, University of South Florida, Tampa, FL 33620, USA; keeyoung@usf.edu (K.Y.H.); thorne3@usf.edu (D.B.); attanayake@usf.edu (S.B.A.); danluu@usf.edu (D.L.); 2 Department of Chemistry and the Texas Center for Superconductivity, University of Houston, Houston, TX 77204, USA; dangminh27498@gmail.com (M.D.N.); trlee@uh.edu (T.R.L.)

**Keywords:** magnetic biosensor, magnetic nanoparticles, magnetic markers, biosensing

## Abstract

Magnetic nanoparticles are extensively utilized as markers/signal labelling in various biomedical applications. Detecting and distinguishing magnetic signals from similarly sized moving magnetic nanoparticles in microfluidic systems is crucial yet challenging for biosensing. In this study, we have developed an original method to detect and differentiate magnetic signals from moving superparamagnetic (SPM) and ferrimagnetic (FM) nanoparticles of comparable sizes. Our approach utilizes a highly sensitive magnetic-coil-based sensor that harnesses the combined effects of giant magnetoimpedance (GMI) and an LC-resonance circuit, offering performance superior to that of conventional GMI sensors. Iron oxide nanoparticles, which have similar particle sizes but differing coercivities (zero for SPM and non-zero for FM) or similar zero coercivities but differing particle sizes, flow through the magnetic coil at controlled velocities. Their distinct effects are analyzed through changes in the complex impedance of the sensing system. Our findings provide a unique pathway for utilizing SPM and FM nanoparticles as innovative magnetic markers to identify specific biological entities, thereby expanding their potential applications.

## 1. Introduction

Magnetic nanoparticles offer diverse applications in biomedicine, spanning imaging, diagnostics, targeted therapy, hyperthermia, and biosensing, thanks to their unique magnetic properties and the ability to tailor them for specific biological interactions [1,2,3,4,5,6,7,8]. Two common types of nanoparticles explored for these applications are superparamagnetic (SPM) and ferrimagnetic (FM) nanoparticles. Unlike FM nanoparticles, which exhibit remanent magnetization and coercivity (*M_r_* ≠ 0, *H_C_* ≠ 0), SPM nanoparticles have zero remanent magnetization and no coercive field (*M_r_* = 0, *H_C_* = 0) [1]. This distinction significantly influences their response to external magnetic fields. While FM nanoparticles tend to aggregate due to strong inter-particle interactions, SPM nanoparticles are non-interacting, preventing aggregation and making them more suitable for biomedical and biosensing applications [6,7,8,9,10,11]. Their superparamagnetic nature also enables a strong magnetic response to external magnetic fields, facilitating the sensitive detection of target biomolecules.

In biosensors and diagnostic assays, magnetic nanoparticles serve as magnetic markers by binding to specific biomolecules (e.g., proteins, DNA, cells) through surface functionalization. This enables selective capture and concentration of target analytes from biological samples [12,13]. Detecting magnetic nanoparticles in microfluidic systems presents challenges but utilizes their magnetic properties and employs sensitive detection methods. Factors like nanoparticle size, shape, composition, and surface coating affect their magnetic behaviors, impacting sensor detection limits, particularly regarding saturation effects [6,7,10,14,15]. Detecting and distinguishing magnetic signals from similarly sized moving magnetic nanoparticles in microfluidic systems is crucial yet challenging for biosensing [16,17,18]. By controlling the flow rate of nanoparticles through the sensor, real-time measurements can provide insights into their concentration and type, allowing for differentiation between SPM and FM nanoparticles based on their unique magnetic signatures.

Various approaches for detecting magnetic nanoparticles have been developed, including magnetic-field-based detection [10], optical detection [19], electrical detection [20], magnetic particle imaging [21], mass-based detection [22], and nuclear magnetic resonance spectroscopy [23]. The choice depends on nanoparticle size, magnetic properties, sensitivity required, microfluidic complexity, and application (e.g., diagnostics, sensing, drug delivery). Integrating these methods into microfluidic platforms allows real-time monitoring, high-throughput analysis, and potential automation [6,7,10,24]. Among these detection methods, magnetic sensors, including Hall effect sensors, magnetoresistive sensors, superconducting quantum interference device (SQUID) sensors, giant magnetoimpedance (GMI) sensors, and inductive coil sensors are particularly effective for detecting the magnetic signals of nanoparticles [8,9,10,25]. Magnetoresistive (MR) sensors have been extensively studied for biosensing applications due to their high sensitivity and compactness [6,8,9]. These sensors detect magnetic signals from magnetic nanoparticles based on the principle of magnetoresistance, which refers to the change in electrical resistance of a material in response to an applied magnetic field. Typically, MR sensors consist of thin film structures with ferromagnetic layers separated by non-magnetic layers [8,9]. The relative orientation of the magnetic moments in these layers determines the sensor’s resistance. When magnetic nanoparticles are in proximity, their magnetic fields influence the alignment of the magnetic moments in the sensor, leading to changes in resistance. These changes are detected as variations in voltage when a constant current flows through the sensor. The output signal corresponds to the presence and characteristics of the nanoparticles: SPM nanoparticles produce fluctuating magnetic fields that affect the sensor’s resistance, while FM nanoparticles create more stable magnetic fields that alter the sensor’s output. While MR biosensors are known for their high sensitivity and compactness, achieving specificity for particular biomolecules or pathogens can be challenging, potentially resulting in false positives [8,14]. Additionally, these sensors are sensitive to temperature variations, which can impact their performance and necessitate careful temperature control during use. They also have a limited dynamic range, which may restrict their effectiveness in detecting a broad spectrum of magnetic signals of nanoparticles in microfluidic sensing systems [14].

Magnetic biosensors leveraging the GMI effect in soft magnetic materials (such as wires, ribbons, or thin films) have recently gained attention for their ultrahigh sensitivity to the magnetic properties of nanoparticles [10,26,27,28,29]. These sensors experience significant changes in AC impedance in response to a magnetic field as nanoparticles move through them, altering the magnetic field and resulting in measurable impedance variations. However, current designs of GMI biosensors struggle to achieve high detection sensitivity for magnetic signals from nanoparticles in microfluidic systems [10]. This limitation arises because detection sensitivity decreases significantly when magnetic nanoparticles are located at a distance from the sensing element, whether it be a wire, ribbon, or thin film.

**Figure 1:**
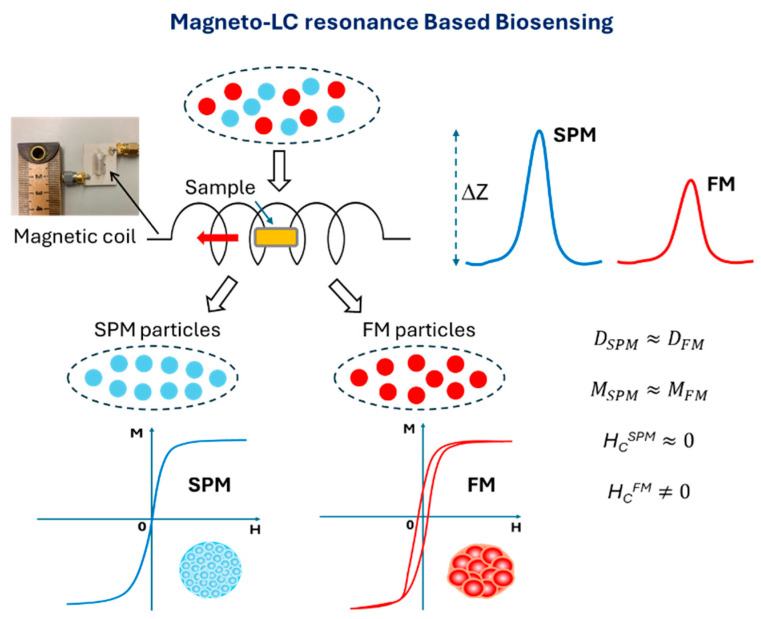
Schematic diagram of nanoparticle detection using a magnetic-coil-based sensor. The sensor distinguishes between superparamagnetic (SPM) and ferrimagnetic (FM) nanoparticles of comparable sizes (*D_SPM_* ~ *D_FM_*) by measuring the impact of their coercivity differences (*H_C_^SPM^* = 0 vs. *H_C_^FM^* ≠ 0) on the sensor’s sensitivity, represented as changes in impedance (Δ*Z*), allowing for the identification of each nanoparticle type. An image of the magnetic coil used in the MLCR sensor is also displayed.

In this context, we developed a novel magnetic-coil-based sensor using soft magnetic microwires, which integrates GMI with LC-resonance circuits, known as magneto-LC-resonance (MLCR) sensor technology [30]. This sensor’s ultrahigh sensitivity and coil-based design hold significant promise for various applications, including industrial process control, healthcare monitoring, and biodetection [30,31,32]. To enhance microfluidic biosensing, we introduce a new method utilizing the MLCR sensor technology (Figure 1) that enables both the detection and the differentiation of magnetic signals from SPM and FM nanoparticles of similar size moving through the sensing coil. Despite similar saturation magnetization, their differing coercivities (zero for SPM nanoparticles, non-zero for FM nanoparticles) can be analyzed via changes in the complex impedance of the sensing system. Our findings offer new avenues for utilizing the distinct properties of SPM and FM nanoparticles as magnetic markers, expanding their potential applications in identifying specific biological entities. An example of how the desired SPM and FM nanoparticles can be employed for detection and recognition of two different antibodies in a microfluidic biosensing system is as follows: The SPM and FM nanoparticles are designed to conjugate with two distinct types of antibodies, AB1 and AB2, respectively. As these nanoparticles flow through a coil channel, one type of antigen is immobilized to selectively recognize one of the two antibodies. By analyzing the signal detected from the magnetic coil as the ferrofluid passes through, we can differentiate between the SPM and FM nanoparticles and identify the corresponding antibodies attached to each.

## 2. Magneto-LC-Resonance (MLCR)-Based Biosensing: Theoretical Considerations

Let us begin by examining the working principle of a conductive magnetic coil, starting with Faraday’s law of electromagnetic induction [33,34].

(1) $$\oint \vec{E}.\vec{ds}=-\frac{d}{dt}\iint \vec{B}.\vec{da}$$

where $\vec{B}$ is the magnetic flux density in the core, $\vec{E}$ is the induced non-conservative electric field, s is the electric lines, and a is the area of the core. For the coil, the time-varying flux density induces a voltage across the ends of the coil, which can be described as

(2) $$V=-nA\frac{d\vec{B}}{dt}$$

where n is the number of turns and A is the cross-sectional area of the core. For the magnetic flux density, we can rewrite $\vec{B}=\mu_{c}\left({\vec{H}}_{t}+\vec{M}\right)$, where $\mu_{c}$ is the magnetic permeability inside the coil, $\vec{M}$ is the magnetization of the magnetic wire, and ${\vec{H}}_{t}$ is the total magnetic field, which is a sum of the ac excitation field ${\vec{H}}_{e}$ and the external dc magnetic field ${\vec{H}}_{dc}$; therefore, the overall field is as follows: ${\vec{H}}_{t}={\vec{H}}_{e}+{\vec{H}}_{dc}$. Since the magnetic sample is moving through the coil, $\mu_{c}$ is also time-variable.

(3) $$V=-nA[\frac{d\mu_{c}}{dt}\left({\vec{H}}_{e}+{\vec{H}}_{dc}+\vec{M}\right)+\mu_{c}\left(\frac{d{\vec{H}}_{e}}{dt}+\frac{d{\vec{H}}_{dc}}{dt}+\frac{d\vec{M}}{dt}\right)]$$

However, the sample’s volume fraction out of the coil is less than 5%, leading to the fact that $\frac{d\mu_{c}}{dt}\left({\vec{H}}_{e}+{\vec{H}}_{dc}+\vec{M}\right)$ is negligible. If the sample has low or zero coercivity, $\frac{d{\vec{H}}_{dc}}{dt}$ is negligible, resulting in the reduced form

(4) $$V=-nA\mu_{c}\left(\frac{d{\vec{H}}_{e}}{dt}+\frac{d\vec{M}}{dt}\right)$$

Using the following expression $\frac{d\vec{M}}{dt}=(\frac{d\vec{M}}{dH})\frac{d{\vec{H}}_{e}}{dt}$, we can rewrite (4) as

(5) $$V=-nA\mu_{c}\left(1+\frac{d\vec{M}}{dH}\right)\frac{d{\vec{H}}_{e}}{dt}$$

where $\frac{d\vec{M}}{dH}$ is the susceptibility of the magnetic wire χ, and 1+χ can be considered as the relative permeability of the magnetic wire $\mu_{w}$.

(6) $$V=-nA\mu_{c}\mu_{w}\frac{d{\vec{H}}_{e}}{dt}$$

with an AC current I and the reactance X=V/I in the coil system, given by X=2πfL, where L is the inductance. Finally, we can determine the inductance using the large length-to-diameter ratio:

(7) $$L\approx \frac{n^{2}\mu_{c}\mu_{w}A}{l}$$

where l is the length of the coil [35,36,37]. When the magnetic sample is positioned inside the coil, it alters the magnetic permeability of the coil, consequently causing variations in the coil’s inductance as the sample travels through it.

We considered a simplified model of a non-ideal coil consisting of inductance (*L*) and resistance (*R_L_*) in series and parasitic capacitance (*C_L_*) in parallel [30]. The complex impedance of the coil ($Z_{coil}$) has two components in series $\left(R_{L}+iwL\right)$ and in parallel to that series $\left(-\frac{i}{wC_{L}}\right)$, where i is the imaginary unit and w is the angular frequency.

(8) Zcoil=11RL+iwL+1−iwCL

Since the magnetic wire is highly conductive, it has very small resistance. As a result, the complex impedance of the coil can be written as

(9) $$Z_{coil}\approx \frac{iwL}{1-w^{2}LC_{L}}$$

**Figure 2:**
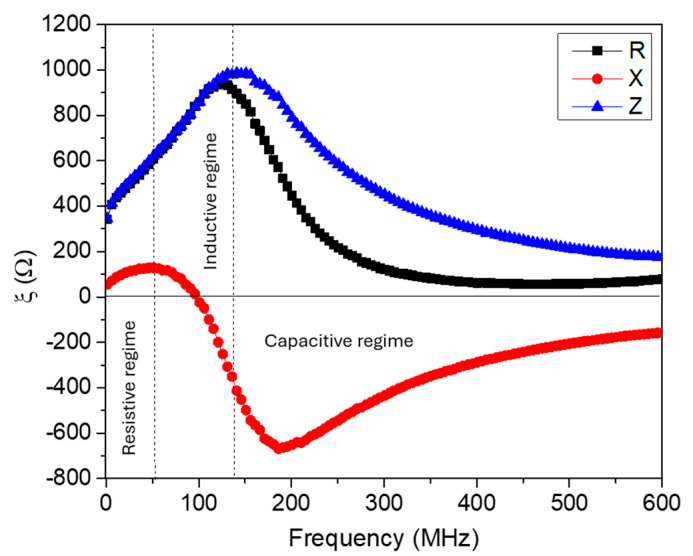
Frequency dependence of the impedance (*Z*), resistance (*R*), and reactance (*X*) of the magnetic coil in the absence of a magnetic field.

The frequency dependence of the complex impedance and its components for the magnetic coil is illustrated in Figure 2.

As depicted in Figure 2, this frequency dependence can be classified into three regimes: resistive, inductive, and capacitive. In the resistive regime (*f*_ac_ ≤ 50 MHz) and the inductive regime (50 < *f*_ac_ ≤ 140 MHz), the measured *R* contributes to *Z* more than *X* does. However, in the capacitive regime for the higher frequency range (*f*_ac_ > 140 MHz), *R* and *X* both significantly contribute to *Z*. It is therefore essential to select an operating frequency that optimizes biosensing, where the presence of magnetic nanoparticles induces the most significant change in the complex impedance.

Recalling that the sensor is constructed from the magnetic wire and operates at a high frequency, approximately a few hundred megahertz, we anticipate a GMI effect when exposed to a small dc magnetic field [38]. In the presence of a magnetic sample with considerable coercivity, which acts as an external small dc magnetic field source, this induces a variation in the magnetic permeability of the magnetic wire and consequently alters its skin depth (*δ*):

(10) $$\delta =\frac{c}{\sqrt{4\pi^{2}f\sigma \mu_{w}}}$$

where c is the speed of light and f is the ac frequency. This change in *δ* affects the resistance and inductance of the magnetic wire through their respective relationships.

(11) $$R_{w}=\frac{\rho l}{2\pi \left(a-\delta \right)\delta }$$

(12) $$L_{w}=\frac{0.175\mu_{0}lf\mu_{w}}{\omega }$$

Both the resistance ($R_{w}$) and inductance ($L_{w}$) of the magnetic wire contribute to the complex impedance ($Z_{wire}$) where the GMI effect occurs. We should therefore consider the change in the total impedance of the system due to the magnetic field, which encompasses alterations in both the coil’s impedance and the wire’s impedance.

(13) $$\Delta Z_{total}=\Delta Z_{coil}+\Delta Z_{wire}$$

where $\Delta Z_{coil}=Z_{coil}\left(0\right)-Z_{coil}\left(S\right)$ and $\Delta Z_{wire}=Z_{wire}\left(0\right)-Z_{wire}\left(S\right)$, with 0 and *S* for the coil/wire impedance without a sample and with a sample, respectively. The total change in impedance (Δ*Z_total_*) serves as a measure of the biosensor’s detection sensitivity.

**Figure 3:**
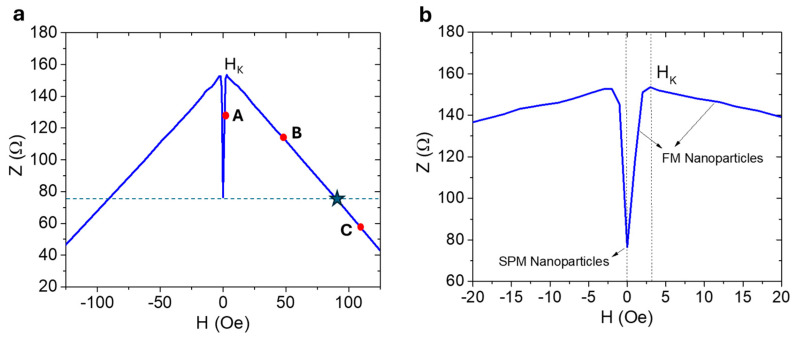
(**a**) The magnetic field dependence of the wire’s impedance at a frequency of 325 MHz, which is the operating frequency of the MLCR sensor. (**b**) A zoomed-in portion of the low-field Z(H) curve. Points A, B, and C represent the three distinct regimes where the presence of magnetic nanoparticles with different coercivities affects the complex impedance of the wire.

According to Equation (13), the presence of a magnetic sample affects the impedance of the system, incorporating changes in both the coil’s impedance and the impedance of the magnetic wire. The large low-field variation in the impedance of the magnetic wire, a phenomenon known as the GMI effect, becomes prominent only when the magnetic sample possesses significant coercivity, whereas the coil’s impedance or inductance varies with both zero and non-zero coercivity values of the magnetic sample. It is worth noting that at high frequencies, the impedance of the magnetic wire is proportional to the square root of its magnetic permeability [38], which varies with external magnetic fields, as illustrated in Figure 3a,b, for a soft magnetic Co_69.25_Fe_4.25_Si_12_B_13.5_Nb_1_ microwire used in our study.

From this figure, we observe that impedance initially increases with increasing magnetic field until it reaches a critical point known as the effective magnetic anisotropy field (*H_K_*). Beyond this point, impedance decreases at higher magnetic fields until it saturates. If the coercive field of the magnetic sample is zero (*H_C_* = 0), as in the case of SPM nanoparticles, no change in the wire impedance is expected (Δ*Z_wire_* = 0), and consequently, the total impedance change (Δ*Z_total_*) is solely influenced by the change in coil impedance (Δ*Z_coil_*). However, when the magnetic sample has a non-zero coercivity value (*H_C_* ≠ 0), as seen in the case of FM nanoparticles, it can act as an external DC magnetic field source. For example, at position A, where *H* < *H_K_*, the presence of the magnetic sample in the coil increases the impedance of the wire (Δ*Z_wire_* < 0), resulting in a negative contribution of Δ*Z_wire_* to Δ*Z_total_*. This means that, compared to the SPM nanoparticles, Δ*Z_total_* decreases when the FM nanoparticles with coercive fields less than the *H_K_* value of the magnetic wire flow through the coil. At position B, where *H_K_* < *H* < *H_cr_* (the critical field above which Δ*Z_wire_* takes a negative sign), the magnetic sample still increases the impedance of the wire with respect to its zero-field state (Δ*Z_wire_* < 0), leading to an overall decrease in the total change in system impedance. Conversely, at position C, where *H_cr_* < *H*, the magnetic sample decreases the impedance of the magnetic wire with respect to its zero-field state (Δ*Z_wire_* > 0), leading to an overall increase in the total change in system impedance. These contrasting behaviors lead us to propose that our sensor can differentiate between two magnetic nanoparticle systems of the same size—those with coercivity (FM nanoparticles) and those without coercivity (SPM nanoparticles)—as well as detect variations in coercivity among FM nanoparticle samples. Furthermore, our sensor can enable real-time tracking of the aggregation behavior of FM nanoparticles, which tend to cluster together rapidly under certain conditions. This capability allows for precise monitoring of nanoparticle dynamics, providing valuable insights into their behavior in complex environments. While the MLCR sensor offers several advantages, such as high sensitivity to weak magnetic fields, non-invasive detection, and effective detection of moving magnetic particles in microfluidic channels across a wide dynamic range, it also has limitations, including circuit design complexity, interference from nearby magnetic objects, and the need for precise design to ensure optimal performance for specific applications. To enhance the sensor’s sensitivity and selectivity, careful attention must be given to factors such as coil geometry, operating frequency, and shielding techniques.

## 3. Experimental Section

### 3.1. Sensor Design, Fabrication, and Testing

The magnetic-coil-based sensor exhibits both the GMI effect and LC-resonance effect due to the Co-rich microwire and its spiral loop shape. This sensor, also named the magneto-LC-resonance (MLCR) sensor, was developed by our group for real-time monitoring of human health [30,31,32]. Details of the design and fabrication of the MLCR sensor are reported in Ref. [30]. In this study, the magnetic coil was constructed from a melt-extracted amorphous ultrasoft magnetic Co_69.25_Fe_4.25_Si_12_B_13.5_Nb_1_ microwire with a 60 µm diameter, wrapped in 15 turns around a plastic tube (diameter: 4 mm; length: 7 mm). The amorphous Co_69.25_Fe_4.25_Si_12_B_13.5_Nb_1_ microwires, fabricated using the melt-extraction technique, demonstrate exceptional magnetic and mechanical properties, making them suitable for fabrication into coil forms. The number of turns in the coil can be adjusted to optimize the sensor’s performance. The design of the MLCR sensor allows magnetic samples to move through the coil easily while enabling the measurement of their effects on the total impedance of the coil. A conventional GMI sensor, consisting of a single straight magnetic microwire with the same Co_69.25_Fe_4.25_Si_12_B_13.5_Nb_1_ composition, was also fabricated to allow for a direct comparison with the performance of the MLCR sensor. It is worth noting that several MLCR and GMI sensors were fabricated to validate the accuracy and consistency of their detection performance. Using the MLCR sensor, we aimed to simulate and detect signals from a microfluidic system containing different types of magnetic nanoparticles, with a particular focus on differentiating the signals of SPM and FM nanoparticles, which exhibit contrasting magnetic behaviors. To achieve this, we adapted a stepper motor to control the sample’s speed through the sensor. As the sample moved through the center of the sensor, its magnetic properties, along with those of the surrounding environment, changed, resulting in a variation in impedance (∆*Z*), as illustrated in Figure 1. All nanoparticle samples were tested under identical conditions, with a sample speed of approximately 2 cm s^−1^ and a mass of 14 mg. The differences in ∆*Z* observed were attributed to the varying magnetic properties of the samples.

### 3.2. Synthesis and Characterization of Magnetic Nanoparticles

The polycrystalline iron oxide (Fe_3_O_4_) nanoparticles were synthesized using solvothermal methods within a binary solvent system of ethylene glycol and diethylene glycol, incorporating modifications in the technical setup and chemical additives. Details of the nanoparticles’ synthesis are reported in [39]. It is noteworthy here that technical parameters such as stirring speed and heating rate can influence nanoparticle size; specifically, increased stirring speed or a using a slower ramping rate can lead to larger nanoparticles. By adjusting these parameters, varying the solvent composition, and altering the amounts of sodium acetate and water, the size of the polycrystalline iron oxide nanoparticles can be fine-tuned from 160 to 400 nm. Additionally, this process allows for variation in their crystallite size from approximately 10 nm in the SPM regime to 20 nm in the FM regime. In this study, we focused on polycrystalline iron oxide nanoparticle samples of similar size (approximately 160 nm), with crystallite sizes varying from 10 nm to 26 nm.

Scanning electron microscopy (SEM) was performed using an FEI Dual Beam 235 Focused Ion Beam system, operating at 15 kV, to image the nanoparticles. Samples were prepared by dissolving them in ethanol and drop-casting onto clean silicon wafers. X-ray diffraction (XRD) data were collected using a Smart Lab system from Rigaku, with Cu Kα irradiation at 40 kV and 44 mA, employing a 0.01° step size for all samples. The crystallite size was determined using the Scherrer formula, based on the diffracted peak from the (311) plane at a 2θ value of 35.5°. Magnetic measurements were conducted using a Physical Property Measurement System (PPMS) from Quantum Design, Inc., equipped with a vibrating sample magnetometer (VSM). The measurements were carried out in the temperature range of 10 to 350 K, with a maximum applied magnetic field of 2 T.

## 4. Results and Discussion

### 4.1. Iron Oxide Superparticles: Superparamagnetism Versus Ferrimagnetism

It has been reported that single crystalline iron oxide (Fe_3_O_4_) nanoparticles can exhibit either superparamagnetic (SPM) or ferrimagnetic (FM) behavior at room temperature, depending on their particle size [11]. There exists a critical size around 15–25 nm below which Fe_3_O_4_ nanoparticles are superparamagnetic and above which they exhibit ferrimagnetic properties. 

To overcome this size limit, we recently synthesized a novel class of SPM superparticles composed of polycrystalline Fe_3_O_4_ nanoparticles with particle sizes ranging between 50 nm and 400 nm while maintaining crystallite sizes of approximately 10 nm within each particle [39]. Since the crystallite size of the SPM superparticles (~10 nm) falls below a certain threshold (~15 nm), the individual magnetic moments of the nanocrystals can no longer maintain long-range magnetic order. Instead, the nanocrystals exhibit superparamagnetism, behaving as if they have a single magnetic domain, with their magnetization easily reorienting in response to an external magnetic field [1,2]. This superparamagnetic behavior is highly advantageous for biosensing applications, as it enables easy manipulation and separation of the nanoparticles using an external magnetic field, without the risk of particle aggregation or loss of magnetic responsiveness over time [40,41]. Additionally, the large surface areas of the SPM superparticles make them particularly beneficial for disease diagnosis and biosensing applications [9,39,42].

Indeed, by harnessing the unique magnetic properties of supercluster particles, with each containing approximately 1000 superparamagnetic iron oxide cores, Kim et al. demonstrated that these nanocluster particles (average size ~ 190 nm) outperform commercially available magnetic nanoparticles in terms of both signal intensity and detection limit for GMR-based biosensing of proteins [9]. In addition, polycrystalline structures offer the advantage of tailoring magnetic properties from SPM to FM without altering particle size simply by adjusting the crystallite size. This approach provides a solution to eliminate particle size effects while enabling investigation of the magnetism effects of magnetic labelling agents in sensing technologies [42,43,44,45].

**Figure 4:**
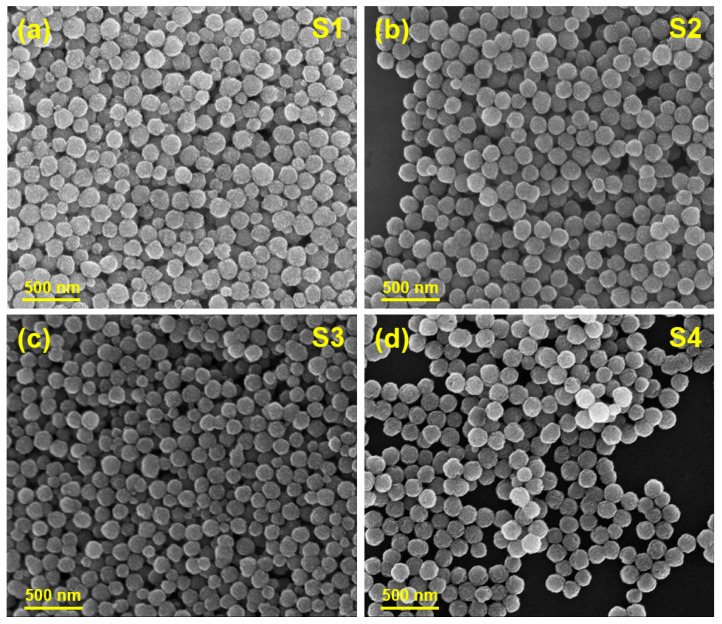
SEM images of (**a**,**b**) the SPM superparticles (S1: *D* = 160 nm, *C_s_* = 10 nm; S2: *D* = 160 nm, *C_s_* = 12 nm) and (**c**,**d**) the FM superparticles (S3: *D* = 160 nm, *C_s_* = 19 nm; S4: *D* = 160 nm, *C_s_* = 26 nm).

**Figure 5:**
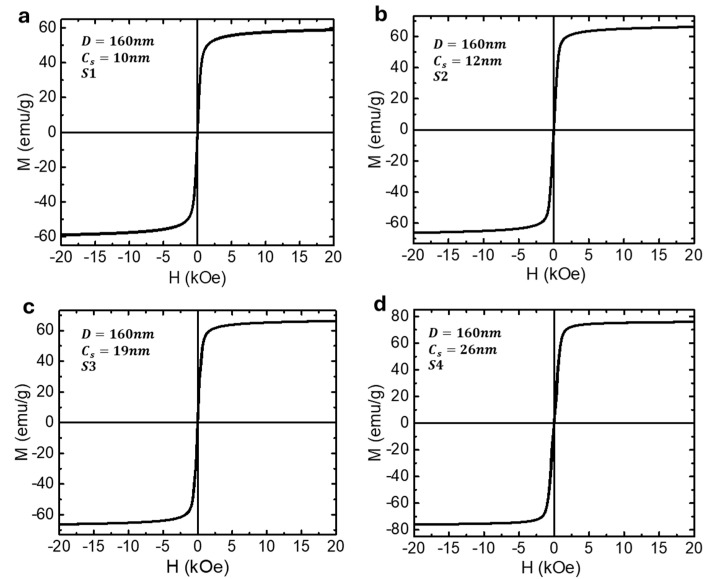
The room-temperature magnetic hysteresis (M-H) loops for (**a**,**b**) SPM superparticles (S1: *D* = 160 nm, *C_s_* = 10 nm; S2: *D* = 160 nm, *C_s_* = 12 nm) and (**c**,**d**) FM superparticles (S3: *D* = 160 nm, *C_s_* = 19 nm; S4: *D* = 160 nm, *C_s_* = 26 nm).

In this study, we focus on Fe_3_O_4_ superparticles of similar overall size (~160 nm) but with varying crystallite sizes (10, 12, 19, and 26 nm). This variation in crystallite size enables us to classify the superparticles into two groups: SPM superparticles (10 and 12 nm) and FM superparticles (19 and 26 nm). They are denoted as samples S1, S2, S3, and S4, respectively. Figure 4 shows the SEM images and Figure 5 shows the corresponding magnetic hysteresis (M-H) data taken at 300 K for these samples. Table 1 provides information about the particle size, crystallite size, and key magnetic parameters of the samples. It is evident from the data that the SPM superparticles (S1 and S2) exhibit zero remanent magnetization and coercive fields, whereas the FM superparticles (S3 and S4) display distinct remanent magnetization and coercivity, confirming their ferrimagnetic behavior. As expected, an increase in crystallite size (*C_S_*) in both SMP and FM cases leads to a corresponding increase in saturation magnetization (*M_S_*).

### 4.2. Biosensing Properties

As described earlier, for biosensing measurements, iron oxide superparticles were compacted inside a plastic sample holder that could be moved through the sensing coil at a controllable speed using a miniature electric motor. To assess the impact of SPM and FM superparticles on the detection sensitivity of the biosensor in microfluidic systems, we monitored changes in the total impedance of the biosensor over time as each magnetic sample (S1, S2, S3, and S4) flowed through the magnetic coil. The change in total impedance (∆*Z_total_*) is used as a measure of the biosensor’s biodetection sensitivity, with accuracy down to milliohms (mΩ). 

As shown in Figure 2, the performance of the MLCR sensor can vary depending on the chosen operating frequency. We evaluated the sensor’s sensitivity for detecting the same sample (S4) at three representative frequencies (resistive, inductive, and capacitive regimes) and found the highest sensitivity in the inductive regime (see Figure S1 in the Supporting Information). Although the detection sensitivity is slightly lower in the capacitive regime compared to the inductive one, the sensing signals in the capacitive regime are more stable. For the purposes of this study, we selected the operating frequency of the sensor in the capacitive regime (*f* = 325 MHz).

**Figure 6:**
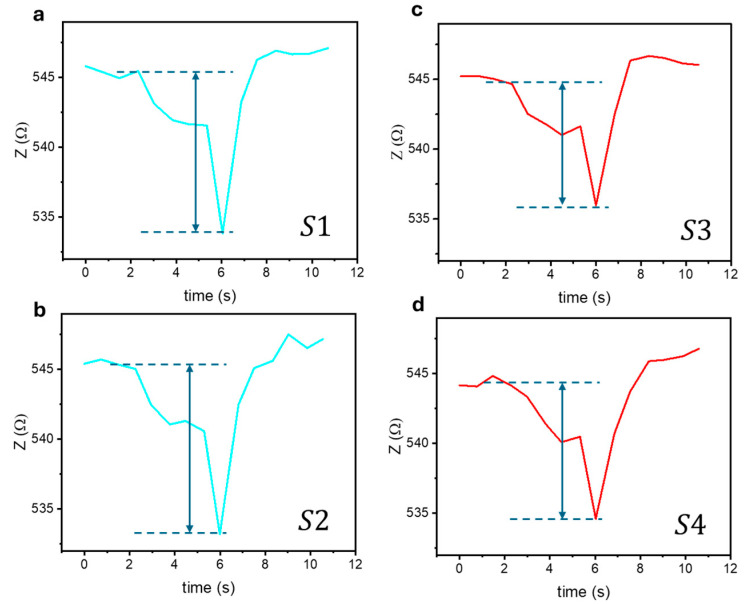
The total impedance of the biosensor varied over time when the iron oxide superparticles of comparable sizes (*D* = 160 nm) flowed through the magnetic coil: (**a**) S1, (**b**) S2, (**c**) S3, and (**d**) S4.

**Figure 7:**
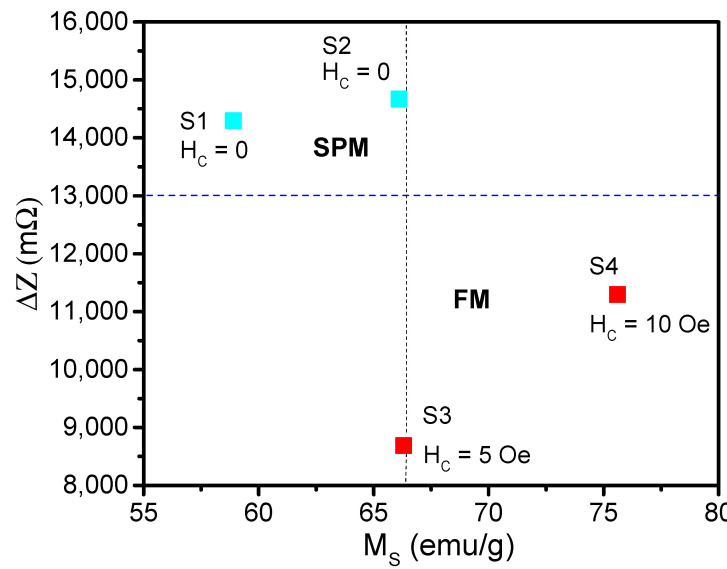
The correlation between the change in total impedance of the biosensor (∆*Z_total_*) and the saturation magnetization (*M_S_*) for the SPM (S1 and S2) and FM (S3 and S4) superparticles highlights a significant difference in ∆*Z_total_* between S2 and S3 despite their identical particle size and saturation magnetization. This demonstrates the biosensor’s ability to differentiate the signals of these two types of nanoparticles.

**Figure 8:**
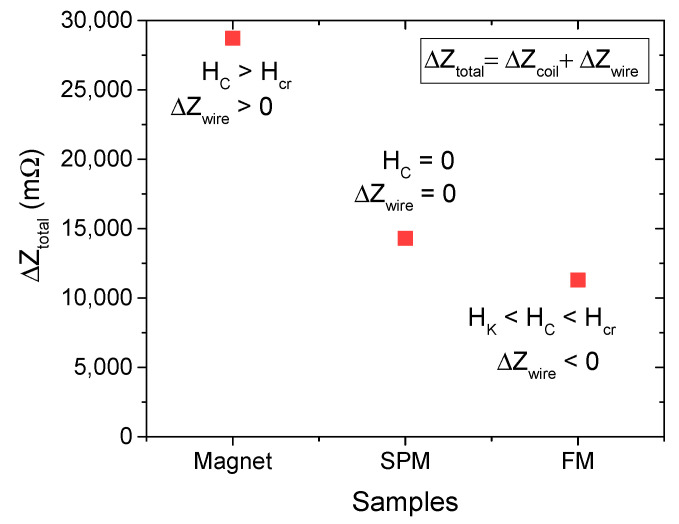
Change in total impedance (∆*Z_total_* or detection sensitivity) for the magnet, SPM (S1), and FM (S4) superparticle samples. The coercivity of the material significantly influences the biosensor’s detection sensitivity.

**Figure 9:**
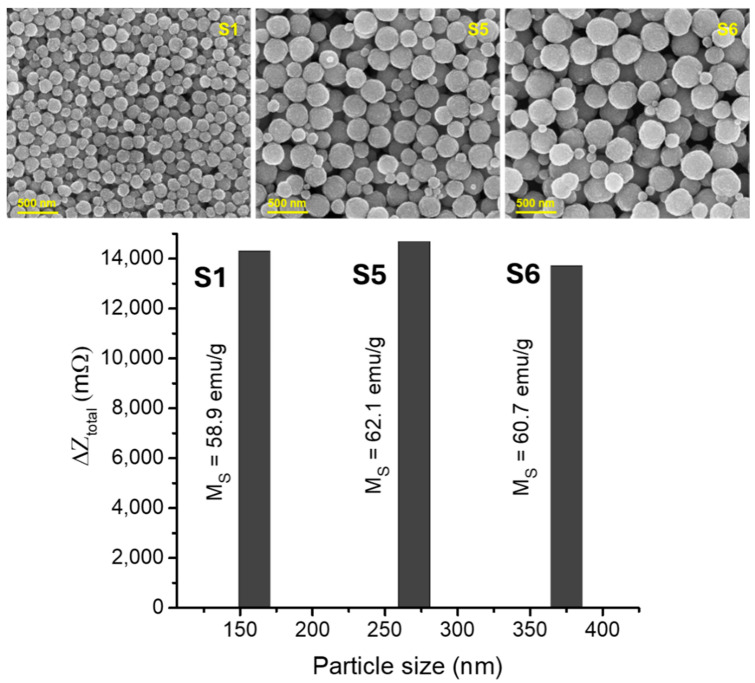
Change in total impedance (∆*Z_total_* or detection sensitivity) versus particle size for the SPM superparticles, which have nearly identical crystallite sizes (~10 nm). The SEM images display the particle sizes and morphologies of the superparticles.

The biosensing results for the different superparticle samples are presented in Figure 6, Figure 7, Figure 8 and Figure 9 and Table 1 and Table 2. As shown in Figure 6, the presence of these superparticles led to significant variations in the total impedance, with changes as large as 14,669 mΩ, demonstrating the ultrahigh sensitivity of the biosensor for detecting magnetic nanoparticles. As anticipated, the MLCR sensor demonstrates significantly superior performance in detecting moving SPM superparticles compared to conventional GMI sensors (see Figure S2 in the Supporting Information). While the GMI sensor’s performance improves for detecting moving FM superparticles, it remains inferior to that of the MLCR sensor (see Figure S3 in the Supporting Information).

For similarly sized SPM superparticles, an increase in crystallite size was found to correlate with higher saturation magnetization [39,46], which in turn led to a greater change in total impedance, reflecting an enhanced detection sensitivity. Specifically, the biodetection sensitivity increased from 14,298 mΩ for sample S1 to 14,669 mΩ for sample S2 as the crystallite size increased from 10 to 12 nm, with corresponding increases in saturation magnetization from 58.9 to 66.1 emu/g.

For FM superparticles of comparable particle sizes, a similar trend was observed but with a more pronounced effect. The biodetection sensitivity significantly increased from 8685 mΩ for sample S3 to 11,294 mΩ for sample S4 as the crystallite size increased from 19 to 26 nm, accompanied by increases in saturation magnetization from 66.3 to 75.6 emu/g and the coercive field from 5 to 10 Oe. Notably, SPM superparticles (S1, S2) exhibited higher detection sensitivities compared to FM superparticles (S3, S4). As illustrated in Figure 7, which highlights the correlation between detection sensitivity and saturation magnetization, samples S2 and S3, despite having nearly identical saturation magnetization values, showed a significant difference in detection sensitivity. This discrepancy arises from the contrasting effects of SPM and FM superparticles, highlighting the biosensor’s ability to differentiate between moving SPM and FM nanoparticles—a crucial feature for microfluidic biosensing applications [47,48].

These findings can be interpreted in the context of the theoretical framework established in Section 3. For SPM superparticles having similar sizes, a higher saturation magnetization leads to a higher detection sensitivity, as it induces a larger variation in inductance and, consequently, the impedance of the coil. This explains why sample S2 exhibited a higher detection sensitivity compared to sample S1. Since SPM superparticles (S1 and S2) exhibit zero coercivity, they have a negligible effect on the impedance of the magnetic wire, resulting in minimal magnetoimpedance effects.

In contrast, FM superparticles (S3 and S4) possess notable coercivity (5 and 10 Oe, respectively), which acts as an external magnetic field, increasing the effective permeability of the magnetic wire and thereby raising the impedance due to the positive magnetoimpedance effect (∆*Z_wire_* < 0). This results in a negative contribution to the ∆*Z_total_*, explaining why the SPM superparticles (S1 and S2) exhibit significantly higher detection sensitivities than the FM superparticles (S3 and S4).

Given the nearly identical particle size and saturation magnetization for samples S2 (SPM) and S3 (FM), the difference in detection sensitivity can be attributed to the contrasting magnetoimpedance effects due to coercivity. While the coercive field in sample S3 does contribute to the impedance change, its effect is less significant compared to the impact of the increased saturation magnetization in sample S4, which leads to a larger change in total impedance and consequently higher detection sensitivity (see Table 1).

We also considered the effect of a magnetic sample (FeNbB) with a much larger coercive field (e.g., a permanent magnet with a coercive field greater than 200 Oe) on the system’s total impedance. As shown in Figure 8, the comparative results for the different samples reveal a significant increase in the total impedance change for the FeNbB sample, which can be attributed to the enhanced magnetoimpedance effect in the magnetic wire.

In this case, the presence of a magnet producing a large DC field (>200 Oe), much greater than the critical magnetic field (~80 Oe) shown in Figure 3, significantly reduces the impedance of the magnetic wire due to the GMI effect, compared to its zero-field state. This leads to a substantial change in the wire’s impedance, which contributes significantly to the total impedance change in the biosensor. In contrast, for the FM superparticles (S4), where the coercive field (10 Oe) is much lower than the critical field (80 Oe), the presence of these particles increases the impedance of the magnetic wire, resulting in a negative contribution to the total impedance change in the biosensor. For the SPM superparticles, however, no contribution to the wire impedance change is observed as their coercive field is zero, leading to no magnetoimpedance effect.

Finally, we examined the effect of SPM particle size on the detection sensitivity of the biosensor by studying SPM superparticles with identical crystallite sizes but different particle sizes: S1 (*D* = 160 nm, *C_S_* = 10 nm), S5 (*D* = 271 nm, *C_S_* = 10 nm), and S6 (*D* = 357 nm, *C_S_* = 10 nm). The details of the particle size, crystallite size, and magnetic properties for these SPM samples, along with their corresponding detection sensitivity values, are summarized in Table 2.

To investigate the relationship between saturation magnetization and detection sensitivity for these SPM superparticles, Figure 9 presents their detection sensitivity values alongside their saturation magnetization data, as well as SEM images depicting their particle sizes and morphologies. Despite the effects of particle size distribution, all samples exhibited similar saturation magnetization and detection sensitivity values, as also observed for other types of SPM superparticles (see Figure S4 in the Supporting Information). This suggests that the particle size of SPM superparticles can be tuned over a broad range—from 160 nm to 375 nm—without compromising the biosensor’s high detection sensitivity, which is advantageous for applications in disease diagnostics and biosensing.

For microfluidic biosensing applications, the coil-based MLCR sensor offers several advantages over conventional wire- and film-based GMI sensors [49,50,51,52], particularly due to its ability to allow nanoparticles to flow through the coil. By tuning the MLCR sensor to operate in the capacitive regime, it can detect both magnetic and dielectric signals from biological systems, such as core/shell nanoparticles with magnetic cores and non-magnetic biomaterial shells—structures commonly used in advanced biomedical applications [51,52]. Future research should focus on exploring this promising new avenue for enhancing biosensing capabilities. 

## 5. Conclusions

In conclusion, our study demonstrates the effectiveness of the MLCR sensor in differentiating between superparamagnetic (SPM) and ferrimagnetic (FM) nanoparticles in simulated microfluidic systems. We found that SPM nanoparticles (S1, S2), with crystallite sizes (10 and 12 nm) smaller than a critical size (~15 nm), exhibited only the LC-resonance effect. In contrast, FM nanoparticles (S3, S4), with larger crystallite sizes (19 and 26 nm), displayed both the LC-resonance and GMI effects. This distinction resulted in a significant difference in the total change in impedance (∆*Z_total_*), which serves as a measure of detection sensitivity, particularly for samples S2 and S3, which have an identical particle size and saturation magnetization but contrasting coercivity values. The performance of the MLCR sensor is shown to outperform that of conventional GMI sensors. These findings highlight that the MLCR sensor can effectively differentiate between SPM and FM nanoparticles of similar size and saturation magnetization, making it well suited for simultaneous detection of these nanoparticles as markers in medical diagnostics and microfluidic biosensing applications.

## Figures and Tables

**Table 1:** Particle size, crystallite size, and key magnetic parameters (*M_s_*, *H_c_*) of the studied samples (S1, S2, S3, and S4). The total impedance change (∆*Z_total_*) is also included for these samples.

Sample	$D\left(nm\right)$	$C_{s}\left(nm\right)$	Msemug	$H_{c}\left(Oe\right)$	$\Delta Z_{total}\left(m\Omega \right)$
S1 (SPM)	160	10	58.9	0	14,298
S2 (SPM)	160	12	66.1	0	14,669
S3 (FM)	160	19	66.3	~5	8685
S4 (FM)	160	26	75.6	~10	11,294

**Table 2:** Particle size, crystallite size, and key magnetic parameters (*M_s_*, *H_c_*) of the SPM samples with the same crystallite size (10 nm) but different particle sizes (S1, S5, and S6). The total impedance change (∆*Z_total_*) is also included for these samples.

Sample	$D\left(nm\right)$	$C_{s}\left(nm\right)$	Msemug	$H_{c}\left(Oe\right)$	$\Delta Z_{total}\left(m\Omega \right)$
S1 (SPM)	160	10	58.9	0	14,298
S5 (SPM)	271	10	62.1	0	14,673
S6 (SPM)	375	10	60.7	0	13,707

## Data Availability

Data are contained within the article and supplementary materials.

## Supplementary Materials

The following supporting information can be downloaded at: https://www.mdpi.com/article/10.3390/bios15020116/s1, Figure S1: Performance of the MLCR sensor at three representative operating frequencies: resistive, inductive, and capacitive regimes; Figure S2: Comparison of the sensitivity between the MLCR sensor and the GMI sensor in detecting the same SPM nanoparticle samples; Figure S3: Comparison of the sensitivity between the MLCR sensor and the GMI sensor in detecting the same FM nanoparticle samples; Figure S4: The change in total impedance (or detection sensitivity) versus particle size for SPM nickel-zinc ferrite (NZF) superparticles with particle sizes of 70 and 107 nm, which have nearly identical crystallite sizes (~8 nm), is shown. The SEM images (a,b) illustrate the particle sizes and morphologies of the 70 and 107 nm superparticles, respectively. Similar detection sensitivities are observed for both of these SPM superparticles (c,d).
