# Supplementary material for: Signal Differentiation of Moving Magnetic Nanoparticles for Enhanced Biodetection and Diagnostics
Source: Biosensors (Basel). 2025 Feb 17;15(2):116. doi: 10.3390/bios15020116 (PMC11852982; doi:10.3390/bios15020116)

Supporting Information

Signal Differentiation of Moving Magnetic Nanoparticles for Enhanced Biodetection and Diagnostics

Kee Young Hwang[1], Dakota Brown[1], Supun B. Attanayake[1], Dan Luu[1], Minh Dang Nguyen[2], T. Randall Lee[2], and Manh-Huong Phan[1,*]

[1]Department of Physics, University of South Florida, Tampa, FL 33620, USA

[2]Department of Chemistry and the Texas Center for Superconductivity, University of Houston, Houston, Texas 77204, USA

*Corresponding author: phanm@usf.edu

Figure S1. Performance of the MLCR sensor at three representative operating frequencies: resistive, inductive, and capacitive regimes.

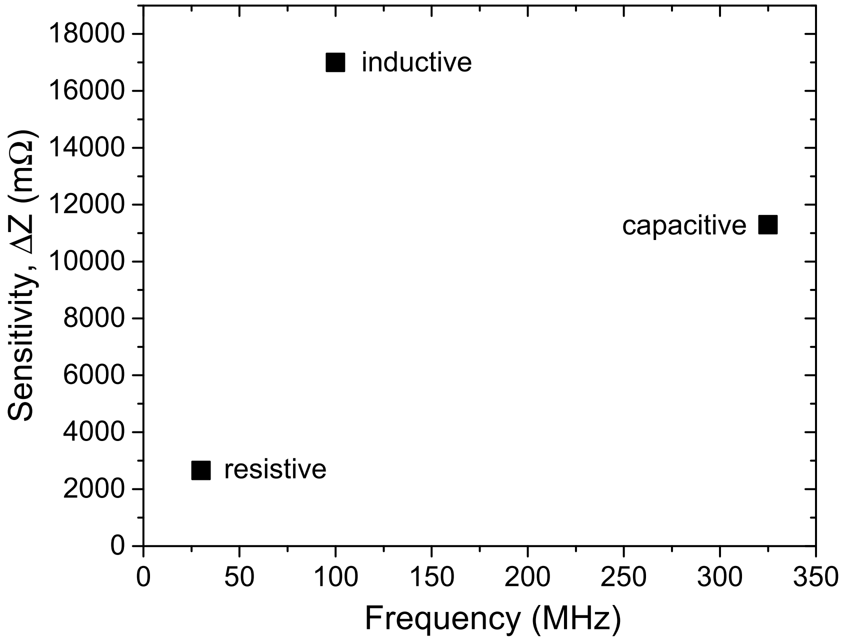

To assess the sensitivity of the MLCR sensor in detecting magnetic nanoparticles at three different frequencies corresponding to the resistive, inductive, and capacitive regimes, as shown in Figure 2, we performed biosensing measurements on sample S4, with the results presented in Figure S1. As expected, the sensor exhibited the highest sensitivity in the inductive regime. However, the sensing signal proved to be most stable in the capacitive regime. For the purposes of this study, we selected the operating frequency of the MLCR sensor in the capacitive regime.

Figure S2. Comparison of the sensitivity between the MLCR sensor and the GMI sensor in detecting the same SPM nanoparticle samples.

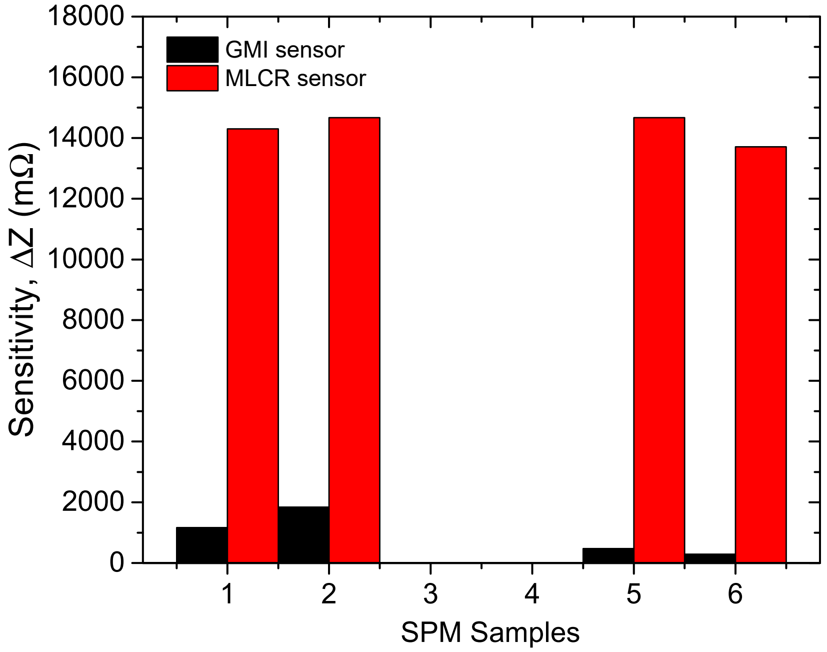

As expected, the GMI sensor shows minimal changes in impedance as the SPM superparticles pass along the microwire. Under the same conditions, the MLCR sensor demonstrates significantly larger changes in impedance, leading to much higher sensitivity. This highlights the clear advantages of the MLCR sensor in detecting SPM particles in microfluidic systems.

Figure S3. Comparison of the sensitivity between the MLCR sensor and the GMI sensor in detecting the same FM nanoparticle samples.

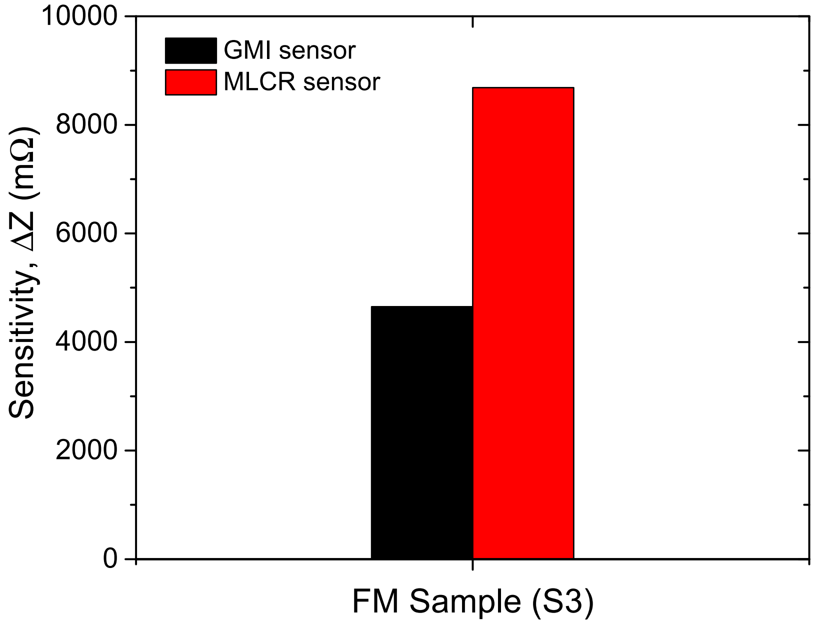

As expected, the GMI sensor shows improved performance in detecting FM superparticles compared to SPM superparticles. However, the detection sensitivity of the GMI sensor remains lower than that of the MLCR sensor.

Figure S4. The change in total impedance (or detection sensitivity) versus particle size for SPM nickel-zinc ferrite (NZF) superparticles with particle sizes of 70 and 107 nm, which have nearly identical crystallite sizes (~8 nm), is shown. The SEM images (a,b) illustrate the particle sizes and morphologies of the 70 and 107 nm superparticles, respectively. Similar detection sensitivities are observed for both of these SPM superparticles (c,d).

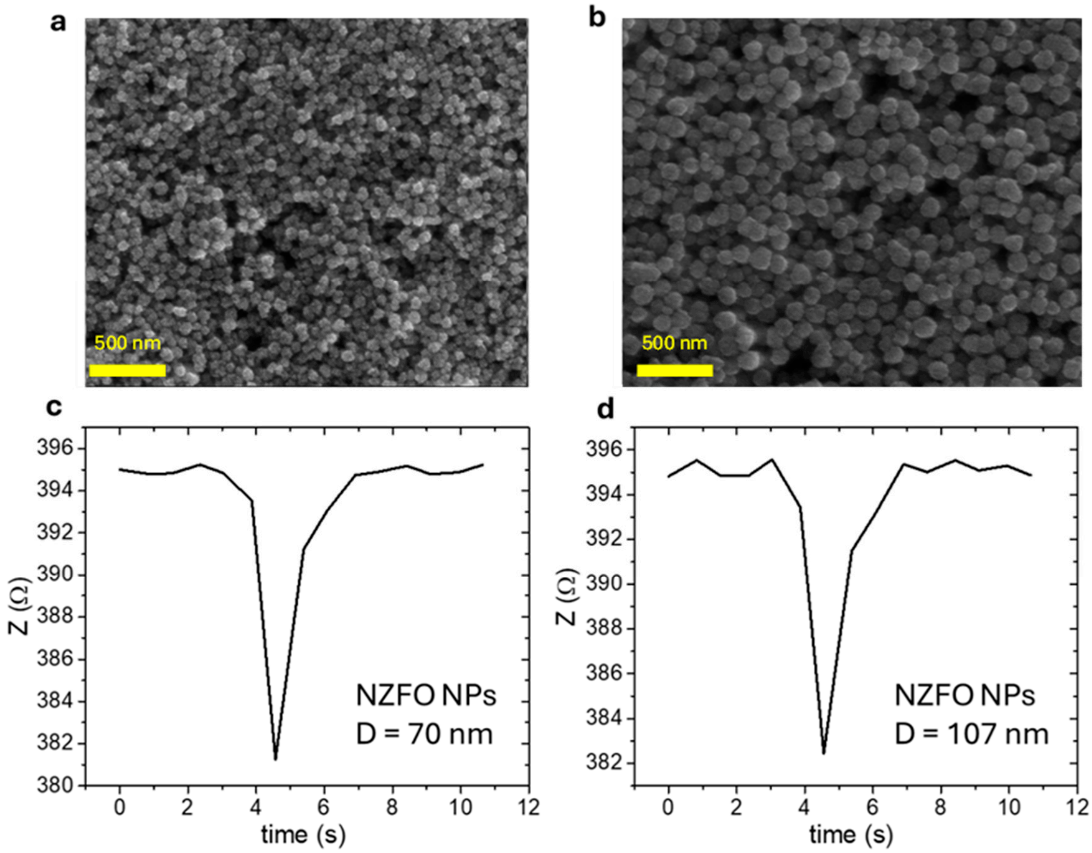

Supplement: Supplementary file 1 [file biosensors-15-00116-s001.zip › biosensors-3385843-supplementary.pdf]
